# Role of mammography accessibility, deprivation and spatial effect in breast cancer screening participation in France: an observational ecological study

**DOI:** 10.1186/s12942-022-00320-5

**Published:** 2022-12-24

**Authors:** Nirmala Prajapati, Patricia Soler-Michel, Verónica M. Vieira, Cindy M. Padilla

**Affiliations:** 1 Univ Rennes, EHESP, CNRS, Inserm, Arènes-UMR 6051, RSMS-U 1309, 35000 Rennes, France; 2 Centre Régional de Coordination des Dépistages des Cancers Auvergne Rhône Alpes, Lyon, France; 3 Department of Environmental and Occupational Health, Program in Public Health, University of California, Irvine, CA USA

**Keywords:** Breast cancer screening, Spatial accessibility, Spillover effect, Deprivation, Spatial autoregressive models

## Abstract

**Background:**

The detection of cancer in its early latent stages can improve patients’ chances of recovery and thereby reduce the overall burden of the disease. Our objectives were to investigate factors (geographic accessibility and deprivation level) affecting mammography screening participation variation and to determine how much geographic variation in participation rates can be explained by spillover effects between adjacent areas, while controlling for covariates.

**Methods:**

Mammography screening participation rates between 2015 and 2016 were calculated by census blocks (CB), for women aged 50–74 years, residing in Lyon metropolitan area. Global spatial autocorrelation tests were applied to identify the geographic variation of participation. Spatial regression models were used to incorporate spatial structure to estimate associations between mammography participation rate and the combined effect (geographic accessibility and deprivation level) adjusting for modes of travel and social cohesion.

**Results:**

The mammography participation rate was found to have a statistically significant and positive spatial correlation. The participation rate of one CB was significantly and positively associated with the participation rates of neighbouring CB. The participation was 53.2% in residential and rural areas and 46.6% in urban areas, p < 0.001. Using Spatial Lag models, whereas the population living in most deprived CBs have statistically significantly lower mammography participation rates than lower deprived ones, significant interaction demonstrates that the relation differs according to the degree of urbanization.

**Conclusions:**

This study makes an important methodological contribution in measuring geographical access and understanding better the combined effect of deprivation and the degree of urbanization on mammography participation and other contextual factors that affect the decision of using mammography screening services -which is a critical component of healthcare planning and equity.

**Supplementary Information:**

The online version contains supplementary material available at 10.1186/s12942-022-00320-5.

## Background

With 2,088,849 new cases and 626,679 deaths globally in 2018, breast cancer is a significant public health problem worldwide [1]. In France in 2018, breast cancer was the most common cancer observed (58,459 new cases) and ranked first among all cancer deaths for women in the country (14,434 deaths), followed by lung cancer and colorectal cancer [2]. The increased mortality risk due to breast cancer can be partly attributed to the barriers to access to early detection and diagnostic services [3], which remain the cornerstone for improving breast cancer outcomes and related survival rates [4].

Mammography screening is one of the most cost-effective breast cancer screening methods for detecting cancer early and reducing breast cancer mortality [5]. In France, the National Mammography Screening Program (NMSP) invites women ages 50–74 years for mammography screening, free of cost, in accredited radiological centers every two years. Screening consists of double reading each negative mammography and immediate further assessment in case of suspicious results [6]. Previous studies in France have found that women screened are 40% less likely to be diagnosed with late-stage breast cancer [7, 8]. Despite that, participation in mammography screening in France was 59% in 2014 [9], 52% in 2016–2017 [6, 9], and 50% in 2018 [10]. According to the French Institute for Public Health Surveillance, rates are below the acceptable participation rate of 70%, which is recommended to keep the program effective enough to reduce mortality [11].

Previous studies have identified several individual factors like health literacy (knowledge, attitudes, beliefs), health-seeking behaviours, availability of a physician [12], and patient education and support, including access barriers [13, 14], influence women’s participation in mammography screening. In addition, important contextual factors are associated with lower mammography screening participation. Studies have demonstrated socioeconomic inequalities in mammography screening, with women residing in low-income neighbourhoods less likely to get screened [6, 13, 15]. Geographic access is also recognized as a crucial factor influencing healthcare utilization and may reinforce disparities in participation in mammography screening [16, 17]. Further, studies have also suggested that other contextual factors like frequent social interaction and social cohesion result in better participation in mammography screening [18, 19].

### Conceptual framework

A previous study by our team identified clusters of low participation in breast cancer screening in urban and peri-urban areas of the Lyon metropolitan area (MA) [6]. A study in Germany by Vogt et al. [20] suggested that spatial spillover effects might explain the geographical clustering of mammography screening participation [6]. By definition, a measurable spillover represents an effect that is spread from sources (neighbouring census blocks [CB]) to a target (an observed CB) through various mechanisms. In this study, we considered geographic proximity and learning/imitation as mechanisms of the spillover effect in Lyon MA.

The concept of geographic proximity allows us to hypothesize that women living in a CB surrounded by other CBs with high mammography screening participation rates will also tend to have a similarly high participation rate. Moreover, we hypothesized that women living near accredited mammography services would have higher screening participation rates. Conversely, women living farther from the services might choose not to participate to avoid long travel distances or to avoid physical pain during mammography screening [21]. Moreover, the literature also suggests the important role of physicians/general practitioners (GPs) in increasing cancer screening participation [22]. Hence, we supposed that in Lyon MA, CBs having a higher number of GPs would have higher participation rates.

The concept of learning and imitation, derived from Bandura’s (1986) social cognitive theory [23] states that an individual can be a responder and a social stimulus of behaviour to others and, at the same time, may learn from observing the actions of others and their results. Learning and imitation can occur through informal communications and social interaction [19, 24] that influence health-seeking behaviour [18, 20, 25]. Applying this concept, we hypothesized that women in one CB would be more likely to participate in screening if women in nearby CBs predominantly participate in mammography screening because of information sharing, learning and imitation. Conversely, they may choose not to participate in screening if most neighbouring women were not screened.

In this context, we aimed to assess whether geographic accessibility (distance to mammography services, the degree of urbanization) influences mammography screening participation rates and to examine the extent of spatial spillover on mammography screening participation rates due to geographic proximity and learning/imitation by applying spatial autoregressive models.

## Methods

### Study setting

A descriptive, cross-sectional ecological study design was employed to assess the geographic variation in mammography screening participation in 2015 and 2016 in Lyon MA. Lyon MA is the third largest MA in the region of Auvergne-Rhône-Alpes in the Eastern region of France. It is composed of 59 municipalities and 510 CBs, with a total population of 1,381,349 inhabitants in an area of 534 km^2^ in 2016. This area was of interest because participation inequalities in mammography screening persist despite previous intervention studies implemented in highly deprived CBs [26].

### Data and methods

#### Data

For this study, we analyzed factors that influence the mammography participation rate. Due to the ecological design of the study, no patients or the public were involved in the study's planning, design, conduct, or reporting. Participation rates were calculated as the percentage of eligible women (50–74 years and invited by NMSP for a free mammography screening) in a CB in 2015–2016. Data were extracted from the NMSP 2015–2016 dataset of the Auvergne Rhone Alpes region. We focused our study on the spatial effects of mammography participation and the spillover sources, including spatial accessibility, deprivation level, modes of travel, and variables for social cohesion.

#### Geographic accessibility

Geographic accessibility factors include the travel distance to the closest accredited mammography service, the degree of urbanization and the density of GPs. To be considered accredited, the radiologists must be registered members of the NMSP. Travel distance was calculated with the Network Analyst function in ArcGIS (version 10.7) using the French road network. The geographic position of each CB is required as input for the spatial analyses, so we derived the position of the centroid (a measure for the geographical center point of a polygon) for each CB using a zonal geometry function in ArcGIS. The distance (kilometers) from each centroid of the CB to the closest accredited mammography service was calculated based on the shortest driving road. Thus, the travel distance is the measure of distance to be traveled by driving to reach the closest mammography service, considering the realistic road network, type of roads, and corresponding driving speeds along with possibilities of driving preferences such as avoiding highways and crossing the bridge.

The density of GPs was used to represent the professional’s advice and guidance for women to participate in the NMSP. The density of GPs was calculated as the number of general practitioners per 100 women invited to participate in mammography screening.

We used an urban–rural index to represent the degree of urbanization. This index was built using the Principal Components Analysis (PCA) method, a statistical technique for reducing the dimensionality of a dataset. The first component of a final PCA with selected variables corresponds to the score of an urban–rural index from residential and rural to urban CBs. Variables included in the PCA were obtained from the CORINE-Land-Cover [27] and French National Institute for Statistics and Economic Studies (INSEE) in the followings domains: housing characteristics, population density, professional mobility (population who work outside of their municipality), residential mobility, and green space. CBs were classified into three classes: urban, peri-urban, and rural (Fig. 2). CBs located in urban areas are characterized by small homes, unstable housing, working within municipalities, and high population density. CBs in rural areas are characterized by large houses with multiple cars, stable housing, working outside municipalities of their residence, green space, and low density.

#### Adjusted confounders

To account for the socioeconomic disparity, we classified CBs by the socioeconomic deprivation level, measured by a deprivation index. This index was defined and evaluated in previous studies that investigated environmental and health inequalities [6, 28], and it was built by performing multiple PCAs to study redundant variables. The first component of a final PCA with selected variables corresponds to the deprivation score. Data on the following domains were obtained from the French national census of 2014 collected from INSEE [28]: employment, single parent family, education, occupation, immigration status, and proportion of social housing. CBs were classified using tertiles as low (− 2.076, − 0.548), medium (− 0.549, 0.133), and high (0.134, 2.994) levels of deprivation according to the distribution of the score.

To account for the mobility of the population inside and across the CBs, we used two variables: the proportion of the population who used a car for their daily mobility and the proportion of people having no access to any transportation (public transport, car or bike). Data on modes of travel for 2014 were also obtained from INSEE [29]. To test the influence of social cohesion on mammography screening participation, we used two variables as a proxy for social cohesion: the proportion of the married population and the proportion of people living alone in a household. The data were derived from INSEE 2016.

### Statistical analysis

To determine whether a significant difference in the outcome and covariates existed between the three groups of the degree of urbanization, non-parametric Kruskal–Wallis tests with a critical p-value of 0.05 were further used. We mapped spatial patterns in the distribution of mammography screening participation rates by CB and then measured the degree of spatial autocorrelation of the dependent and independent variables by calculating the Global Moran’s I [30]. Briefly, the Global Moran’s I falls between − 1 and 1; a positive Moran’s I value indicates a positive spatial autocorrelation—that the nearby areas have similar values (i.e., clustered)—and a negative value indicates a negative spatial autocorrelation—that the nearby areas have different values (i.e., dispersed). To calculate Moran’s I statistic, we defined a contiguity matrix W using queen continuity weights to define the neighbourhood structure to indicate whether or not CBs share a common boundary.

In this study, we were primarily interested in mammography participation rate patterns that can be explained by the geographic accessibility (i.e., travel distance, density of GPs, degree of urbanization with the rural CBs as references) or/and the deprivation level. To assess the contribution of these factors, we first applied ordinary least squares models adjusted with these factors alone (OLS1), adjusted for mobility and social cohesion (OLS2), and with the interaction between deprivation and degree of urbanization (OLS3). Because observations associated with spatial units may reflect measurement error, spatial autocorrelation was tested on the residuals of each OLS1, OLS2 and OL3 models using the Global Moran’s I [30]. The global Moran’s *I* was statistically significant (p < 0.05) suggesting the necessity to account for the spatial configuration of the CBs in the study (Additional file 1: Table S1).

If spatial autocorrelation was detected, a spatial autoregressive model is required to avoid violating the OLS assumption of independence between features and to ensure that our estimates are unbiased [13, 31]. The spatial autoregressive model incorporates a diffusion process across the geographical location in which the participation rates in a CB are affected by explanatory variables in the same CB and the adjacent ones, and at the same time is also influenced by the participation rates in the adjacent CBs [32].

To decide which spatial autoregressive model is more appropriate, we used the decision rule suggested by Florax and Rey [33] based on the Lagrange Multiplier tests and their robust counterparts by Anselin [34]. Briefly, the choice of model depends on the significance of LAG or error models and their robust forms: robust LM-lag and robust LM-error. If LM-lag is statistically significant and LM-error is not, then Spatial Lag Model (SLM) is appropriate and not the Spatial Error Model (SEM) model. Conversely, if LM-error is statistically significant and LM-lag is not, then the appropriate specification is a SEM and not a SLM model. We conducted diagnostics and the LM-lag of the mammography participation model was statistically significant (p < 0.001) and the LM-error was not (p > 0.05). Therefore, we selected a SLM for subsequent analyses (Additional file 2: Table S2).

An SLM assumes that there is a spatial dependence in the dependent variable, whereas SEM assumes that there is a spatial dependence in the error term. An SLM can be expressed as Eq. (1):

1 $$y = \rho Wy + {\rm X}\beta + \varepsilon$$

2 $$\varepsilon \sim N\left( {0, \sigma^{{2}} In} \right)$$

with *y* as the dependent variable (participation rate), *W* denotes the spatial weight matrix and *ρWy* is the spatially *y* value. *β* is a vector of coefficients of the explanatory variables *X*. The error term, *ε*, follows a normal distribution with a mean 0 and a variance *σ*^*2*^*In,* where *In* is a *n x n* identity matrix.

We first perform adjusted analyses with geographic accessibility factors (i.e., travel distance, density of GPs, degree of urbanization with the rural CBs as references) and the deprivation level alone (SLM1), adjusted for mobility and social cohesion (SLM2), and with the interaction between deprivation and degree of urbanization (SLM3). After each spatial model, the Akaike Information Criterion (AIC) and the pseudo-adjusted coefficient of determination (adjusted R^2^) were used as a goodness of fit estimation for model comparison. We tested the residuals of each spatial lag model for spatial autocorrelation using Moran’s I statistics. We chose a significance level of 0.05 to determine statistical significance. Statistical analyses were performed using R version 4.2.1 (2022-06-23). All GIS processes and map layouts were performed using ArcMap v.10.5 (ESRI, Redlands, CA, USA).

## Results

**Fig. 1:**
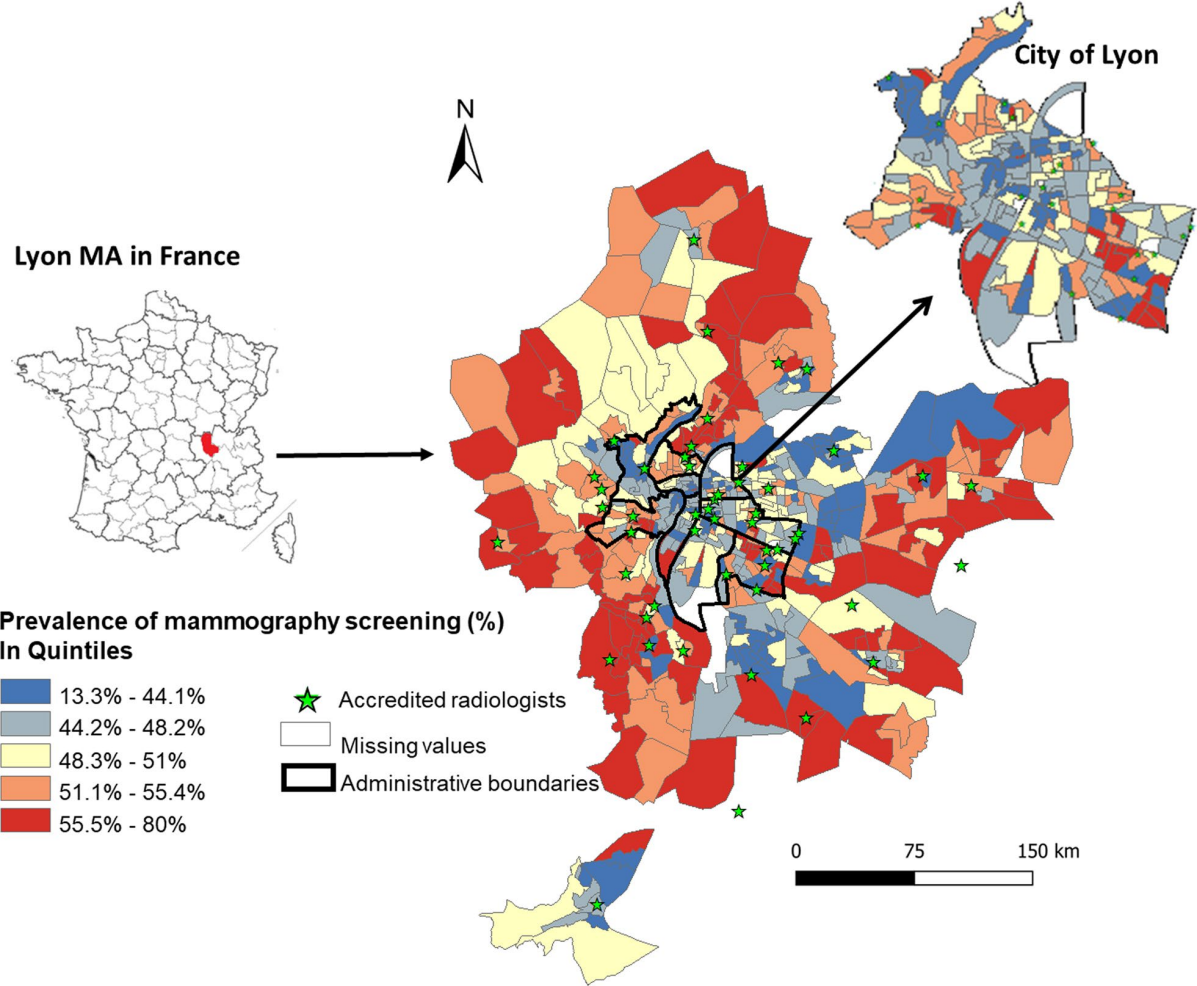
Mammography participation rates in Lyon metropolitan area (2015–2016) by quintiles

**Fig. 2:**
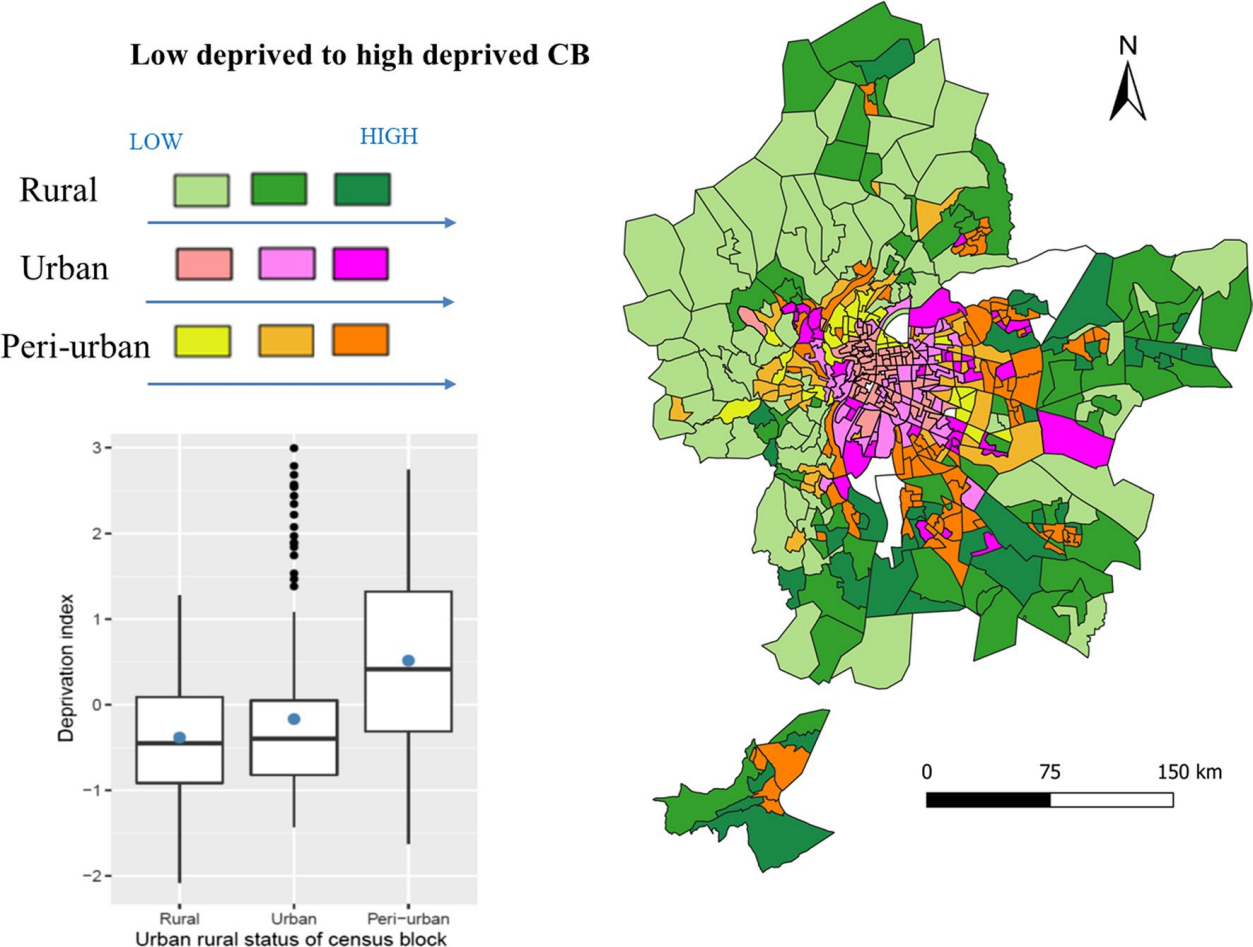
Description with graph and map of the combined effect of urban–rural index and deprivation classes of the census blocks (CB) in the Lyon metropolitan area

In the Lyon MA, in 2015–2016, the NMSP invited a total of 178,002 women aged 50–74 years for mammography screening, but only 88,909 women participated, resulting in the average CB-level participation rate of 48.3% ranging from 13.3% in Venissieux to 80.0% in Lyon 1st district. Geographical distribution showed higher participation indicated by red shades in the rural areas (mean = 53.1%, standard deviation [SD] = 6.16) whereas lower participation in urban (mean = 46.6%, SD = 7.54) and peri-urban (mean = 47.9%, SD = 6.57) areas are indicated by blue shades (Table 1; Fig. 1). Moreover, this map highlights an unequal repartition of mammography services (represented by a green triangle) primarily located in city centers. The mean travel distance differed according to where the population lives (0.98 km in urban, 1.29 km in peri-urban, and 2.30 in rural CBs; p < 0.001). The population with the highest deprivation primarily lived in the peri-urban CBs, whereas, the population with the least deprivation lived in urban and rural CBs (Table 1; Fig. 2). The p-value for a Kruskal–Wallis test to compare the mean deprivation level between the urbanization classes was statistically significant (p < 0.001).

**Table 1:** Description of study variables and their spatial autocorrelation stratified by urban–rural context and overall census blocks

Variables	Overall (n = 496)		Urban	Peri-urban	Rural	p-value
	Mean (SD)	Moran’s *I*	Mean (SD)	Mean (SD)	Mean (SD)	
Screening participation (%)	48.3 (9.90)	0.29 (< 0.001)	46.6 (7.54)	47.9 (6.57)	53.1 (6.16)	< 0.001
**Spatial accessibility**						
Travel distance to the closest mammography (in km)	1.53 (1.15)	0.57 (< 0.001)	0.98 (0.67)	1.29 (0.75)	2.30 (1.42)	< 0.001
Density of GPs^a^	1.12 (1.27)	0.06 (0.01)	1.15 (1.64)	0.73 (0.84)	0.49 (0.77)	< 0.001
**Traveling**						
Traveling by car (%)	53.4 (20.5)	0.74 (< 0.001)	33.7 (10.1)	56.0 (10.0)	73.2 (8.84)	< 0.001
With no transportation access (%)	3.08 (2.19)	0.49 (0.03)	3.40 (1.73)	3.08 (1.48)	3.71 (3.59)	0.13
**Social cohesion**						
Living alone in a household (%)	10.6 (3.95)	0.48 (< 0.001)	27.7 (9.27)	18.0 (5.45)	12.0 (5.20)	< 0.001
Married (%)	41.9 (13.3)	0.54 (< 0.001)	31.1 (10.2)	42.2 (5.68)	51.3 (8.52)	< 0.001
**Socioeconomic deprivation**		0.49 (< 0.001)				< 0.001
High deprivation [n (%)]	168 (33.9)		36 (21.81)	92 (56.79)	40 (23.81)	
Medium deprivation [n (%)]	164 (33.1)		63 (38.19)	40 (24.69)	60 (35.71)	
Low deprivation [n (%)]	163 (32.9)		66 (40.00)	30 (18.52)	68 (40.48)	

*CB* census blocks, *GP* general practitioners, *SD* standard deviation

^a^Per 100 women invited to screen

The global spatial correlation test indicated the presence of the spatial correlation of mammography participation rates in Lyon MA. The geographical variation in participation was identified with a positive and statistically significant Moran’s *I* (0.29; p < 0.001) indicating a positive autocorrelation of the participation rates meaning that neighbouring CBs have similar mammography participation rates. Other contextual variables were also spatially autocorrelated (see Table 1).

Table 2 summarizes estimates of spatial lag models, which measured the spatial spillover of mammography participation and analyzed the spillover sources (geographic accessibility, deprivation, mobility, and social cohesion factors). All SLM models have accounted for spatial autocorrelation; Moran’s I of the SLM model residuals are non-significant. Moreover, the adjusted R^2^ (between 0.4 and 0.45) demonstrates that the spatial lags models explain 40–45% of the variability of the mammography participation rate.

**Table 2:** Models to estimate mammography participation according to the geographic accessibility of services adjusted on covariates

	SLM1	SLM2		SLM 3
	β-coefficient (95%CI)	β-coefficient (95%CI)		β-coefficient (95%CI)
Travel distance continuous (in km)	− 0.49 (− 1.00, 0.01)†	− 0.51 (− 1.01, 0.00)*		− 0.42 (− 0.94, 0.10)
Density of GPs	− 0.12 (− 0.56, 0.32)	− 0.11 (− 0.54, 0.31)		− 0.13 (− 0.55, 0.30)
Degree of urbanization				
Rural	Ref.	Ref.		Ref.
Urban	− 4.58 (− 6.08, − 3.08)**	− 1.42 (− 3.94, 1.10)		− 1.32 (− 3.84, 1.20)
Peri-urban	− 1.66 (− 3.11, − 0.21)*	− 0.31 (− 2.01, 1.39)		− 0.42 (− 2.12, 1.29)
Deprivation level	− 2.81 (− 3.39, − 2.24)**	− 3.19 (− 3.79, − 2.60)**		− 2.98 (− 3.86, − 2.07)**
Interaction terms				
Rural and deprivation				Ref.
Urban and deprivation				− 1.62 (− 3.18, − 0.07)*^a^
Peri-urban and deprivation				− 0.64 (− 2.09, 0.81)
Modes of travel				
% of the population traveling by car		0.13 (0.07, 0.19)**		0.13 (0.07, 0.19)**
% of the population with no transportation access		− 0.24 (− 0.45, − 0.03)*		− 0.24 (− 0.45, − 0.03)*
Social cohesion				
% of the married population		− 0.11 (− 0.19, − 0.05)**		− 0.10 (− 0.17, − 0.02)*
% of the population living alone in a household		− 0.05 (− 0.14, 0.04)		− 0.04 (− 0.13, 0.05)
Moran’s *I*	− 0.04	− 0.04		− 0.04
R^2^	0.40	0.44		0.44
AIC	3134	3103		3102
Spatial lag coefficients				
Participation lag (*ρ*)	0.46 (0.34, 0.59)**	0.38 (0.24, 0.51)**		0.41 (0.27, 0.54)**

*AIC* Aikake’s Information Criterion, *CI* confidence interval, *GP* general practitioner, *km* kilometer

^a^The total effect takes into account the main effect of deprivation and the effect of interaction term urban & deprivation compare to rural CBs. For instance, in SLM3 − 2.98 + (− 1.62) = 4.60

**(p < 0.01), *(p < 0.05), †(p < 0.10)

There was a statistically significant and positive spatial diffusion of participation rates across the CBs. All the SLM models demonstrated statistically significant and positive spatial lag coefficient (*ρ*) for participation rates between 0.46 (SLM1) and 0.41 (SLM 3) after adjusting for mobility and social cohesion (p < 0.001). The participation rate in a CB increased by 0.46% (ρ in SLM1) when the participation rates of neighbouring CBs increased by one percent.

When considering geographic accessibility factors, before adjustment, the degree of urbanization was significantly associated with participation rates; on average, the rural CBs participated more, in mammography screening (β = 1.66; 95%CI: 0.21, 3.11), and the urban CBs participated less compared to the peri-urban CBs(β = − 2.92; 95%CI: − 4.25, − 1.59). This relation disappeared after adjusting for personal mobility by car and social cohesion.

The levels of deprivation of the CBs had a negative and statistically significant effect on the mammography participation rate. There was a statistically significant negative association between the level of deprivation and mammography participation rates. This association remained stable after adjustment on mobility and social cohesion. Significant (p < 0.05) results were found for interaction terms between the level of deprivation and the degree of urbanization in the SLM3 model. The model indicated that the effect of CBs’ deprivation level on mammography participation rates differed by the degree of urbanization. For urban census blocks, one unit increase in the level of deprivation was associated with, an average decrease of mammography participation by 4.30% (− 4.30 correspond to the effect of the level of deprivation − 2.98 and interaction term urban and deprivation − 1.32) compared to a decrease of 2.98% for rural census blocks.

## Discussion

This paper aimed to examine some important determinants of mammography participation and to investigate the main factors affecting its variation and the sources of spatial spillover in Lyon MA. Using data from the NMSP, this study contributed to improving the understanding of spatial accessibility and can help broaden the scope of screening programs.

The results call attention to the presence of spatial spillover in the mammography participation rates across neighbouring CBs in Lyon. With positive spatial lag coefficients for the participation rates, it suggested a spatial diffusion process for the mammography participation rates across the CBs. We demonstrated that the participation rate in one observed CB is significantly and positively influenced by the participation rates of neighbouring CBs. There was no evidence of a spillover effect due to social cohesion. In contrast, previous literature suggested that information, experience and beliefs about the efficiency of screening and individual preferences could be disseminated through informal communication when individuals live in the same or nearest CB and visit the same healthcare workers [20].

Contrary to expectations, the degree of urbanization was inversely associated with mammography participation. We found that women in urban and peri-urban CBs had a lower participation rate than women in rural CBs. A recent Australian study observed similar results [35], whereas several other studies reported a negative association between distance and mammography screening participation [16, 36, 37]. We demonstrated that access to a car for daily mobility is a significant predictor, and after adjusting for this variable, the degree of urbanization and the travel distance were no longer associated with mammography participation.

Our study confirmed that the level of deprivation of the CBs remained a major barrier to mammography participation. High-deprived CBs had 10% lower participation rates than the low-deprived CBs. This finding supported the results of previous studies in France [9, 19], Europe [38], and Canada [36]. Factors that explained this association was well established as low education, ownership of their residence, employment, or low income [39]. In addition, non-native French speakers, who were likely to be immigrants and have lower economic status, may also have additional language and cultural barriers to mammography services [40]. The evidence from previous studies showed that attitudes, behaviour and psychological factors (including fear and anxiety about mammography screening for various reasons) could adversely impact participation rates [9].

Despite deprivation being a crucial contextual factor, its negative influence on mammography screening participation was higher for urban census blocks than rural ones. This paradoxical situation between urban and rural CBs in Lyon could be attributed to the combined effect of deprivation and the importance of mobility. This finding was supported by previous research which showed that rural areas, where people are used to having high mobility for all of their daily activities including commuting and accessing facilities, also have a higher tendency to participate in mammography screening services [41]. This could imply that women living in a highly deprived CB either have limited access to private transportation or the public transportation routes are not favourable enough, and as a result, their movement is geographically confined within the CB. In England, Wang et al. [42] noted the importance of the mobility and relevance of daily transport to improve mammography screening coverage. A previous study in Paris, by Vallée et al. in 2010 demonstrated that unequal participation in preventive healthcare in urban areas may not be directly associated with the spatial distribution of the services, with a notable exception for people with limited activity space for example, for a highly deprived population who cannot afford to travel by car [43]. Similarly, in France, Rican et al. [44] indicated that low residential mobility due to inappropriate urban planning for pedestrians could curb participation rates.

These findings can provide evidence for improving the uptake of mammography screening services. Future interventions could focus on highly deprived CBs where the percentage of the population with a car for daily mobility is very low. Our study did not find a significant role of GPs in mammography participation, which was inconsistent with the role of GPs in encouraging women to prevention and screening [45]. Additionally, it could be relevant to explore the influence of other factors, such as appropriate urban planning for pedestrians in urban areas, waiting for delays, and other individual and service-related factors on mammography participation rates. Similarly, the importance of the location of health services and the associated social dynamics of the neighbourhood could bring relevant understanding to the variation of mammography participation rates.

Strengths of this study include analysis of the geographic distribution of breast cancer screening participation rates using models that accounted for spatial lag. Although the use of GIS tools to visualize the spatial distribution of cancer is well documented [45], only a few studies [12, 20] have successfully captured the effect of the spatial distribution of cancer screening. The travel distance indicator using a road network in this present study is a more realistic indicator than the euclidean distance. The use of a road network considers the real life speed of travel, and the possibilities of choice of roads (avoiding a highway, taking a bridge etc.), whereas, the Euclidean distance considers only the perpendicular geographical distance to the destination. This study made an essential methodological contribution to assessing the geographical access to mammography screening services.

One of the major limitations of this study was the ecological study design and the bias due to ecological fallacy that could not be ruled out despite careful design and execution. Only variables available at the CB level were used in this study; unfortunately, other important socio-demographic information such as race and ethnicity were not available. The results must be interpreted cautiously since the inferences drawn on the CB level may not reflect all the individual-level associations between participation and the explanatory variables. The findings could have been more robust if both individual-level and CB-level data could be used. In addition, our analyses were limited to data from 2015 to 2016. Recent data would have provided a more up-to-date estimate of variation in mammography screening participation in Lyon.

## Conclusion

Given this study’s analysis, the following conclusions can be drawn. Territorial inequalities of mammography participation across Lyon MA persists, despite implementing measures to promote breast cancer screening. The spillover effect is a critical phenomenon to consider in understanding the full impact of interventions at the population level. The impact of geographic accessibility varies according to the level of deprivation of the CB. This study highlighted areas with the significant combined effect of travel distance to mammography services and deprivation, which can help to prioritize them as the areas that need intervention. We illustrated that for areas with high deprivation, lower travel distance is critical to improving the mammography participation. Additional research is needed to assess the role of GPs in delivering preventive care and providing an appropriate recommendation for breast cancer screening. Moreover, further studies could focus on additional individual and service-related factors, e.g., the waiting delay, that could be important in urban areas.

## Supplementary Information

**Additional file 1: Table S1. **Global Moran’I statistics and goodness of fits.

**Additional file 2: Table S2**. Diagnostics for spatial regression modelling.

## Data Availability

All Breast Cancer Screened women consented to their data being used for service improvement.

## Abbreviations

AIC: Akaike information criterion
CB: Census block
GIS: Geographic information system
GP: General practitioners
INSEE: National Institute of Statistics and Economics Studies
LM: Lagrange multiplier
MA: Metropolitan area
NMSP: National Mammography Screening Program
R^2^: Coefficient of determination
SEM: Spatial error model
SD: Standard deviation
SLM: Spatial lag model
