# Supplementary material for: Role of mammography accessibility, deprivation and spatial effect in breast cancer screening participation in France: an observational ecological study
Source: Int J Health Geogr. 2022 Dec 24;21:21. doi: 10.1186/s12942-022-00320-5 (PMC9789573; doi:10.1186/s12942-022-00320-5)
Supplement: Supplementary file 1 — Additional file 1: Table S1. Global Moran’I statistics and goodness of fits. [file 12942_2022_320_MOESM1_ESM.pptx]

## Slide 1
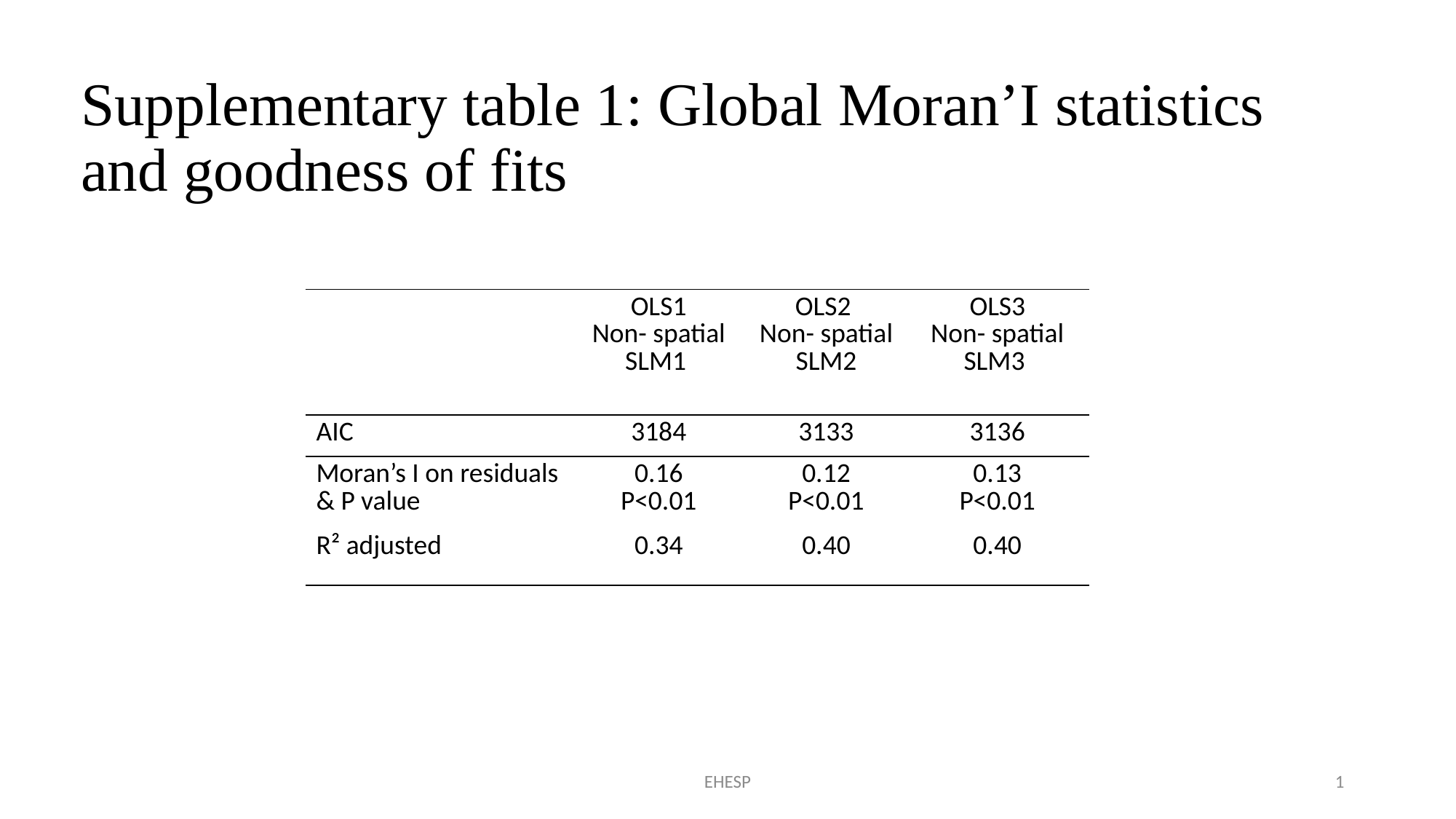

# Supplementary table 1: Global Moran’I statistics and goodness of fits
| | OLS1 Non- spatial SLM1 | OLS2 Non- spatial SLM2 | OLS3 Non- spatial SLM3 |
| --- | --- | --- | --- |
| AIC | 3184 | 3133 | 3136 |
| Moran’s I on residuals & P value | 0.16 P<0.01 | 0.12 P<0.01 | 0.13 P<0.01 |
| R² adjusted | 0.34 | 0.40 | 0.40 |
1
EHESP
