# Supplementary material for: Role of mammography accessibility, deprivation and spatial effect in breast cancer screening participation in France: an observational ecological study
Source: Int J Health Geogr. 2022 Dec 24;21:21. doi: 10.1186/s12942-022-00320-5 (PMC9789573; doi:10.1186/s12942-022-00320-5)
Supplement: Supplementary file 2 — Additional file 2: Table S2. Diagnostics for spatial regression modelling. [file 12942_2022_320_MOESM2_ESM.pptx]

## Slide 1
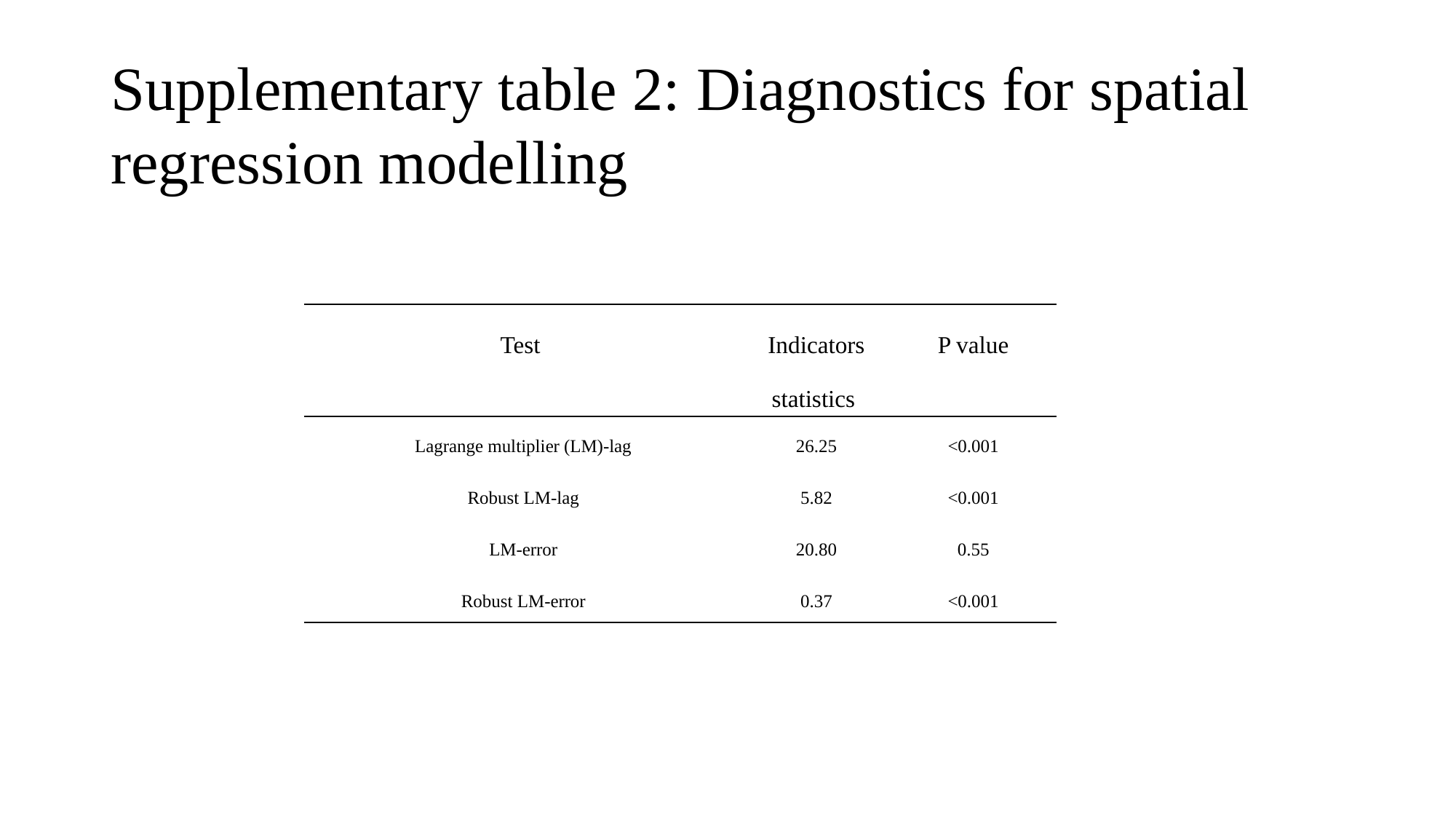

# Supplementary table 2: Diagnostics for spatial regression modelling
| Test | Indicators statistics | P value |
| --- | --- | --- |
| Lagrange multiplier (LM)-lag | 26.25 | <0.001 |
| Robust LM-lag | 5.82 | <0.001 |
| LM-error | 20.80 | 0.55 |
| Robust LM-error | 0.37 | <0.001 |
